# Prioritizing extracellular miRNA candidates in asthma: transparent *in silico* integration, literature-based concordance, and mechanistic network analysis

**DOI:** 10.3389/falgy.2026.1856862

**Published:** 2026-08-05

**Authors:** Ourania S. Kotsiou, Irene Tsilioni, Nikolaos A. A. Balatsos, Konstantinos I. Gourgoulianis, Zoe Daniil, Erasmia Rouka

**Affiliations:** 1 Laboratory of Human Pathophysiology, Department of Nursing, University of Thessaly, Larissa, Greece; 2 Department of Respiratory Medicine, University of Thessaly, Larissa, Greece; 3 Department of Immunology, Tufts University School of Medicine, Boston, MA, United States; 4 Department of Biochemistry and Biotechnology, University of Thessaly, Larissa, Greece; 5 Department of Nursing, University of Thessaly, Larissa, Greece

**Keywords:** asthma, biomarkers, extracellular vesicles, *in silico* analysis, literature-based concordance, miRNA, network analysis, Reactome

## Abstract

**Background:**

Extracellular microRNAs (miRNAs) are promising asthma biomarker candidates, but database-derived panels require literature assessment.

**Objective:**

To prioritize extracellular asthma-associated miRNAs and evaluate literature-based concordance, specificity and target-network structure.

**Methods:**

RNADisease v4.0, miEAA, miRPathDB 2.0, miRDB v6.0, MSigDB v7.4 C3 MIR:MIRDB, Reactome, and Gene Ontology were integrated. Formal concordance required an exact mature-arm identifier and a clearly interpretable asthma contrast in a separate published human extracellular-miRNA study; arm-unspecified names were retained only as contextual evidence. Predicted targets were analyzed using unique MIR:MIRDB target-set counting, Reactome over-representation testing, and Benjamini–Hochberg correction across 1,217 pathways.

**Results:**

Sixty-three extracellular candidates were identified; 60 had an EV/exosome/microvesicle annotation and 53 had at least two localization/transport annotations. Three published studies supported six formally concordant candidates, while four additional candidates had only arm-unspecified contextual support. GSE280322 showed no exact mature-arm overlap. Raw-read reprocessing was feasible but not undertaken; this represented published-result concordance rather than sample-level validation. Fifty-nine candidates mapped to 57 unique target sets and 7,092 genes; four were unmapped, including formally concordant hsa-miR-126-3p, so concordance and network sets were non-identical. The analysis yielded 108 hubs and one FDR-significant, parameter-contingent Reactome pathway: regulation of MECP2 expression and activity.

**Conclusion:**

This framework supports prioritization, but no validated or asthma-specific signature was established.

## 1 Introduction

Asthma remains a major global health challenge affecting individuals of all ages. Its substantial and persistent burden, together with marked clinical and molecular heterogeneity, supports the need for biomarkers that can complement clinical phenotyping and help generate mechanistically informed hypotheses (1).

Recent research has increasingly focused on non-coding RNAs in asthma (2, 3). Circulating miRNAs may be encapsulated in extracellular vesicles (EVs) or associated with proteins such as Argonaute and lipoproteins. EV-associated miRNAs are protected from degradation and can reflect intercellular signaling, making them attractive candidates for disease stratification and treatment-response studies. Nevertheless, differences in biospecimen type, EV isolation, assay platform, normalization, asthma phenotype, and miRNA-arm annotation have limited reproducibility across studies (2, 3).

A further limitation of many *in silico* studies is that they aggregate curated database entries without a transparent and auditable search for additional published evidence or an explicit analysis of shared target-network structure. We therefore aimed to: (i) identify asthma-associated extracellular miRNAs using a transparent multi-resource workflow; (ii) assess exact mature-arm concordance in separate published human cohorts or public datasets; (iii) expand the downstream analysis to predicted miRNA–target hubs and Reactome pathways; and (iv) distinguish candidate generation from clinical biomarker validation.

## 2 Materials and methods

### 2.1 Candidate identification and extracellular localization

We conducted a multi-step *in silico* analysis. The fuzzy-search function of RNADisease v4.0 (http://www.rnadisease.org/) (4) was used to retrieve miRNAs supported by experimental evidence in asthma, yielding 101 accessions (75 unique miRNAs). Subcellular localization was assessed with miEAA 2023 (5), focusing on extracellular, circulating, microvesicle, extracellular-vesicle, and exosomal annotations. Because this permissive rule retained 63/75 candidates (84%), stricter descriptive checks were added: 60/63 candidates carried a specific EV/exosome/microvesicle annotation and 53/63 had at least two retrieved localization/transport annotations. These checks were used to characterize annotation strength, not to redefine the primary panel. For candidates meeting the primary criterion, Table 1 retains the full standardized localization/transport annotation set returned by the source resource, which may also include intracellular compartments; therefore, the annotation does not prove EV cargo, distinguish vesicular from Ago2/HDL-bound miRNA, or identify the cellular source. The exploratory miRPathDB 2.0 candidate-Reactome annotation export (6) was removed from the main manuscript because it was non-inferential; its provenance and interpretive boundary are retained in the Supplementary Data workbook. The original stringent target analysis used miRDB v6.0 (7, 8) predictions with a score of 100, followed by Gene Ontology molecular-function analysis (9, 10). RNADisease batch search was used to retrieve strong-association scores.

**Table 1:** Asthma-associated miRNA candidates with reported localization/transport annotations and literature-based evidence classification.

No.	miRNA	Annotation source	No. unique annotations	Reported localization/transport annotations	Evidence code
1	hsa-miR-1-3p	exRNA forms (miRandola)	2	Circulating; microvesicle	NC
2	hsa-miR-106a-3p	exRNA forms (miRandola)	1	Exosome	NC
3	hsa-miR-122-5p	exRNA forms (miRandola)	4	Microvesicle; Ago2; circulating; exosome	TC
4	hsa-miR-124-3p	exRNA forms (miRandola)	2	Circulating; exosome	CU
5	hsa-miR-125b-5p	exRNA forms (miRandola)	3	Microvesicle; circulating; exosome	TC
6	hsa-miR-126-3p	exRNA forms (miRandola)	2	Circulating; exosome	TC
7	hsa-miR-1296-3p	Localization (RNALocate)	3	Nucleus; mitochondrion; exosome	NC
8	hsa-miR-1307-3p	Localization (RNALocate)	8	Microvesicle; nucleolus; extracellular vesicle; nucleus; exosome; cytoplasm; mitochondrion; circulating	NC
9	hsa-miR-130a-3p	exRNA forms (miRandola)	3	Ago2; circulating; exosome	CU
10	hsa-miR-133b	exRNA forms (miRandola)	2	Circulating; exosome	LU
11	hsa-miR-139-3p	exRNA forms (miRandola)	2	Circulating; exosome	NC
12	hsa-miR-142-3p	exRNA forms (miRandola)	3	Microvesicle; circulating; exosome	NC
13	hsa-miR-145-3p	Localization (RNALocate)	3	Circulating; microvesicle; exosome	NC
14	hsa-miR-146a-5p	exRNA forms (miRandola)	3	Ago2; circulating; exosome	TC
15	hsa-miR-146b-3p	Localization (RNALocate)	5	Microvesicle; nucleus; cytoplasm; circulating; exosome	NC
16	hsa-miR-148a-3p	exRNA forms (miRandola)	3	Ago2; circulating; exosome	NC
17	hsa-miR-149-3p	exRNA forms (miRandola)	1	Exosome	NC
18	hsa-miR-152-3p	exRNA forms (miRandola)	2	Circulating; exosome	NC
19	hsa-miR-155-3p	Localization (RNALocate)	1	Microvesicle	CU
20	hsa-miR-15a-3p	Localization (RNALocate)	1	Exosome	NC
21	hsa-miR-16-5p	exRNA forms (miRandola)	4	Microvesicle; Ago2; circulating; exosome	NC
22	hsa-miR-181a-3p	exRNA forms (miRandola)	1	Exosome	NC
23	hsa-miR-182-3p	exRNA forms (miRandola)	2	Circulating; exosome	NC
24	hsa-miR-182-5p	exRNA forms (miRandola)	2	Circulating; exosome	NC
25	hsa-miR-185-3p	Localization (RNALocate)	3	Circulating; microvesicle; exosome	NC
26	hsa-miR-187-3p	Localization (RNALocate)	3	Microvesicle; exosome; mitochondrion	NC
27	hsa-miR-18a-3p	exRNA forms (miRandola)	1	Circulating	NC
28	hsa-miR-191-5p	exRNA forms (miRandola)	3	Microvesicle; circulating; exosome	TC
29	hsa-miR-192-3p	exRNA forms (miRandola)	1	Circulating	NC
30	hsa-miR-19b-3p	exRNA forms (miRandola)	5	Microvesicle; Ago2; microparticle; circulating; exosome	NC
31	hsa-miR-200a-3p	exRNA forms (miRandola)	2	Circulating; exosome	NC
32	hsa-miR-200b-3p	exRNA forms (miRandola)	4	Microvesicle; Ago2; circulating; exosome	NC
33	hsa-miR-203a-3p	exRNA forms (miRandola)	2	Circulating; exosome	NC
34	hsa-miR-20a-5p	exRNA forms (miRandola)	4	Microvesicle; Ago2; circulating; exosome	NC
35	hsa-miR-21-3p	exRNA forms (miRandola)	3	Microvesicle; circulating; exosome	NC
36	hsa-miR-216a-3p	Localization (RNALocate)	1	Exosome	NC
37	hsa-miR-22-3p	exRNA forms (miRandola)	4	Microvesicle; Ago2; circulating; exosome	NC
38	hsa-miR-221-3p	exRNA forms (miRandola)	4	Microvesicle; Ago2; circulating; exosome	CU
39	hsa-miR-223-3p	exRNA forms (miRandola)	5	Microvesicle; Ago2; circulating; HDL; exosome	TC
40	hsa-miR-26a-1-3p	Localization (RNALocate)	4	Nucleus; mitochondrion; microvesicle; exosome	NC
41	hsa-miR-27a-3p	exRNA forms (miRandola)	4	Microvesicle; Ago2; circulating; exosome	NC
42	hsa-miR-27b-3p	exRNA forms (miRandola)	3	Ago2; circulating; exosome	NC
43	hsa-miR-28-5p	exRNA forms (miRandola)	2	Circulating; exosome	NC
44	hsa-miR-29b-3p	exRNA forms (miRandola)	4	Microvesicle; Ago2; circulating; exosome	NC
45	hsa-miR-29c-3p	exRNA forms (miRandola)	4	Microvesicle; Ago2; circulating; exosome	NC
46	hsa-miR-30a-3p	exRNA forms (miRandola)	1	Exosome	NC
47	hsa-miR-31-5p	exRNA forms (miRandola)	3	Microvesicle; circulating; exosome	NC
48	hsa-miR-326	exRNA forms (miRandola)	2	Circulating; exosome	LU
49	hsa-miR-331-3p	exRNA forms (miRandola)	2	Circulating; exosome	NC
50	hsa-miR-33a-3p	Localization (RNALocate)	2	Microvesicle; exosome	NC
51	hsa-miR-33b-3p	Localization (RNALocate)	2	Microvesicle; exosome	NC
52	hsa-miR-374a-3p	exRNA forms (miRandola)	2	Circulating; exosome	NC
53	hsa-miR-375-3p	exRNA forms (miRandola)	5	Microvesicle; Ago2; circulating; HDL; exosome	NC
54	hsa-miR-376a-3p	exRNA forms (miRandola)	3	Ago2; circulating; exosome	NC
55	hsa-miR-423-3p	exRNA forms (miRandola)	3	Microvesicle; circulating; exosome	NC
56	hsa-miR-483-3p	exRNA forms (miRandola)	2	Circulating; exosome	NC
57	hsa-miR-485-3p	Localization (RNALocate)	4	Mitochondrion; circulating; microvesicle; exosome	NC
58	hsa-miR-495-3p	Localization (RNALocate)	3	Microvesicle; mitochondrion; exosome	NC
59	hsa-miR-508-3p	exRNA forms (miRandola)	1	Circulating	NC
60	hsa-miR-590-5p	exRNA forms (miRandola)	2	Circulating; exosome	NC
61	hsa-miR-625-5p	exRNA forms (miRandola)	2	Circulating; exosome	NC
62	hsa-miR-629-3p	Localization (RNALocate)	2	Microvesicle; exosome	NC
63	hsa-miR-98-5p	exRNA forms (miRandola)	2	Circulating; exosome	NC

Literature-based evidence codes: TC, formal exact mature-arm concordance in a separate published human extracellular-miRNA study; CU, contextual evidence reported only with an arm-unspecified miRNA name and therefore not counted as an exact mature-arm match; NC, no exact mature-arm concordance identified in the audited literature-based evidence set; LU, legacy arm-unspecified panel identifier excluded from the arm-resolved sensitivity analysis. The six TC candidates are hsa-miR-122-5p, hsa-miR-125b-5p, hsa-miR-126-3p, hsa-miR-146a-5p, hsa-miR-191-5p, and hsa-miR-223-3p. For TC candidates, no exact overlap was observed with the published differential-miRNA lists for the reported GSE280322 contrasts. Annotation terms were standardized for capitalization and exact case-insensitive duplicates were counted once. The reported annotation list may include intracellular localizations recorded by RNALocate alongside the extracellular or transport-related annotation that qualified the candidate. Bold miRNA names indicate TC candidates. Ago2, Argonaute 2; HDL, high-density lipoprotein.

Legacy exploratory outputs are consolidated in the Supplementary Data workbook. They were not used to define hubs, select pathways, or support inferential conclusions. Supplementary Figure S1 instead presents the corrected auditable search, linkage, evidence-classification, and analysis workflow. The duplicate-aware, multiplicity-controlled MIR:MIRDB-Reactome workflow described below remains the primary network analysis.

### 2.2 Auditable search for additional datasets and studies

On 12 June 2026, we searched NCBI GEO, SRA/BioProject, EMBL-EBI BioStudies/ArrayExpress, PubMed/MEDLINE, and Europe PMC. Detailed search strategies, eligibility criteria, screening and deduplication procedures, and sensitivity analyses are provided in the Supplementary Appendix and Supplementary Data workbook. Raw live-interface totals and complete original exports were not retained and are therefore not retrospectively reconstructed; accordingly, the search is described as transparent and auditable rather than fully reproducible from inception. For the retained auditable set, 23 records entered detailed review (7 repository records and 16 publication records). Consolidation of three linked repository-publication pairs yielded 20 study-level units; 6 were excluded and 14 were retained in the evidence audit (1 public-dataset published-result assessment, 3 formal literature-concordance studies, and 10 contextual studies). Records were deduplicated by accession, PMID, DOI, and normalized title. Evidence was classified as formal exact mature-arm concordance, arm-unspecified contextual evidence, contextual-only evidence without candidate-level exact matching, or exclusion, with identifier-resolution status and record-level reasons tabulated in the Supplementary Data workbook.

### 2.3 Literature-based evidence assessment

GSE280322/PRJNA1177844 and its associated publication (11) were identified as potentially suitable for sample-level assessment. A standard raw-read reanalysis using a fully specified small-RNA sequencing pipeline was technically feasible; however, *de novo* sample-level differential-expression analysis was not undertaken in the present study. GEO provides a normalized-count matrix and links to raw SRA reads, but reprocessing would require explicit choices regarding adapter trimming, reference release, alignment, feature quantification, quality-control thresholds, and normalization. To avoid presenting a newly constructed *post hoc* pipeline as a reproduction of the original analysis, normalized values were not treated as raw counts, rounded, or converted into pseudo-counts. We therefore performed only a dataset-level exact mature-arm concordance assessment using the differentially abundant miRNAs reported for the comparison-specific contrasts in the associated publication. This assessment is presented as published-result concordance, not as sample-level validation or replication. Formal literature-based concordance required a separate published human extracellular or cell-free miRNA study, an exact mature-arm-resolved -3p/-5p identifier, a clearly defined asthma comparator, phenotype, or severity contrast, and a direction-specific or significance-specific group-level result. Availability of a reusable sample-level matrix was not required. Arm-unspecified names such as miR-124, miR-130a, miR-155, and miR-221 were not mapped to a specific -3p/-5p panel member and were retained only as contextual evidence. Exact matching was harmonized to miRBase v22.1 nomenclature; opposite arms, arm-unspecified identifiers, and different family members were not considered equivalent. A manual source-overlap check was attempted for all six formally concordant candidates, but complete candidate-level RNADisease source-study provenance was unavailable in the accessible export; independence therefore could not be verified for any candidate. Literature-based concordance is consequently treated as a prioritization signal rather than independent replication.

### 2.4 Expanded target-network and pathway analysis

The MSigDB v7.4 C3 MIR:MIRDB collection (12) was used for expanded MIR:MIRDB predicted target-set mapping; reference (12) is retained as the canonical MSigDB database citation, whereas the specific release used for the analysis was v7.4. Candidate names were mapped at the mature-arm level where specified. Two MSigDB sets jointly represented miR-27a-3p/miR-27b-3p and miR-29b-3p/miR-29c-3p; therefore, the primary analysis counted each unique target set once, preventing duplicate inflation of gene degree. Candidate-level counting was retained only as a sensitivity analysis. Genes targeted by at least seven unique target sets were defined operationally as primary hubs, with thresholds of ≥6 and ≥8 examined in sensitivity analyses. The 10–500 background-gene Reactome pathway-size filter, the ≥7 hub threshold, and unique-set counting were fixed before the final duplicate-aware pathway run during revision and are therefore described as analysis-defined rather than prospectively prespecified. Reactome over-representation analysis used the 16,668 MIR:MIRDB genes as background. One-sided hypergeometric *p* values were calculated for all 1,217 eligible Reactome pathways, followed by Benjamini–Hochberg correction across the complete tested family. FDR < 0.05 was considered significant. A second sensitivity analysis excluded the arm-unspecified legacy identifiers hsa-miR-133b and hsa-miR-326. Database vintages are reported together to avoid over-interpretation across mixed releases: RNADisease v4.0, miEAA 2023, miRPathDB 2.0, miRDB v6.0, MSigDB v7.4, Reactome as exported from MSigDB v7.4, and the 2026 Gene Ontology release used only for the exploratory molecular-function provenance result.

## 3 Results

### 3.1 Candidate identification

RNADisease v4.0 yielded 101 asthma-associated miRNA accessions, corresponding to 75 unique miRNAs. Integration with miEAA extracellular-localization annotations identified 63 extracellular candidates (Table 1). The permissive extracellular filter retained 84% of the disease-associated set. Under stricter descriptive checks, 60/63 candidates retained a specific EV/exosome/microvesicle annotation, while 53/63 had at least two retrieved localization/transport annotations. These results show that the extracellular filter enriched for miRNAs with extracellular or transport-related annotations but did not localize the source or prove vesicular cargo. RNADisease strong-association scores are provided in Table 2. These 63 miRNAs constitute a candidate panel rather than a measured expression signature.

**Table 2:** RNADisease strong-association scores and batch-export status for the 63 extracellular candidate miRNAs.

miRNA	RNA type	Species	Disease	Association score
hsa-miR-1-3p	miRNA	Homo sapiens	Asthma	0.999945
hsa-miR-106a-3p	miRNA	Homo sapiens	Asthma	NR
hsa-miR-122-5p	miRNA	Homo sapiens	Asthma	0.90146
hsa-miR-124-3p	miRNA	Homo sapiens	Asthma	0.973352
hsa-miR-125b-5p	miRNA	Homo sapiens	Asthma	0.968971
hsa-miR-126-3p	miRNA	Homo sapiens	Asthma	0.973352
hsa-miR-1296-3p	miRNA	Homo sapiens	Asthma	0.999999
hsa-miR-1307-3p	miRNA	Homo sapiens	Asthma	0.999999
hsa-miR-130a-3p	miRNA	Homo sapiens	Asthma	NR
hsa-miR-133b	miRNA	Homo sapiens	Asthma	0.694506
hsa-miR-139-3p	miRNA	Homo sapiens	Asthma	0.99998
hsa-miR-142-3p	miRNA	Homo sapiens	Asthma	0.999831
hsa-miR-145-3p	miRNA	Homo sapiens	Asthma	0.694506
hsa-miR-146a-5p	miRNA	Homo sapiens	Asthma	0.90146
hsa-miR-146b-3p	miRNA	Homo sapiens	Asthma	1
hsa-miR-148a-3p	miRNA	Homo sapiens	Asthma	0.994838
hsa-miR-149-3p	miRNA	Homo sapiens	Asthma	1
hsa-miR-152-3p	miRNA	Homo sapiens	Asthma	0.998598
hsa-miR-155-3p	miRNA	Homo sapiens	Asthma	1
hsa-miR-15a-3p	miRNA	Homo sapiens	Asthma	0.999141
hsa-miR-16-5p	miRNA	Homo sapiens	Asthma	0.999831
hsa-miR-181a-3p	miRNA	Homo sapiens	Asthma	0.973352
hsa-miR-182-3p	miRNA	Homo sapiens	Asthma	0.999141
hsa-miR-182-5p	miRNA	Homo sapiens	Asthma	0.999999
hsa-miR-185-3p	miRNA	Homo sapiens	Asthma	NR
hsa-miR-187-3p	miRNA	Homo sapiens	Asthma	0.694506
hsa-miR-18a-3p	miRNA	Homo sapiens	Asthma	0.694506
hsa-miR-191-5p	miRNA	Homo sapiens	Asthma	NR
hsa-miR-192-3p	miRNA	Homo sapiens	Asthma	NR
hsa-miR-19b-3p	miRNA	Homo sapiens	Asthma	0.999831
hsa-miR-200a-3p	miRNA	Homo sapiens	Asthma	1
hsa-miR-200b-3p	miRNA	Homo sapiens	Asthma	1
hsa-miR-203a-3p	miRNA	Homo sapiens	Asthma	0.99998
hsa-miR-20a-5p	miRNA	Homo sapiens	Asthma	0.973352
hsa-miR-21-3p	miRNA	Homo sapiens	Asthma	1
hsa-miR-216a-3p	miRNA	Homo sapiens	Asthma	NR
hsa-miR-22-3p	miRNA	Homo sapiens	Asthma	0.994838
hsa-miR-221-3p	miRNA	Homo sapiens	Asthma	0.998598
hsa-miR-223-3p	miRNA	Homo sapiens	Asthma	1
hsa-miR-26a-1-3p	miRNA	Homo sapiens	Asthma	0.968971
hsa-miR-27a-3p	miRNA	Homo sapiens	Asthma	0.694506
hsa-miR-27b-3p	miRNA	Homo sapiens	Asthma	0.999999
hsa-miR-28-5p	miRNA	Homo sapiens	Asthma	0.90146
hsa-miR-29b-3p	miRNA	Homo sapiens	Asthma	0.998598
hsa-miR-29c-3p	miRNA	Homo sapiens	Asthma	NR
hsa-miR-30a-3p	miRNA	Homo sapiens	Asthma	1
hsa-miR-31-5p	miRNA	Homo sapiens	Asthma	0.90146
hsa-miR-326	miRNA	Homo sapiens	Asthma	1
hsa-miR-331-3p	miRNA	Homo sapiens	Asthma	NR
hsa-miR-33a-3p	miRNA	Homo sapiens	Asthma	0.994838
hsa-miR-33b-3p	miRNA	Homo sapiens	Asthma	1
hsa-miR-374a-3p	miRNA	Homo sapiens	Asthma	0.694506
hsa-miR-375-3p	miRNA	Homo sapiens	Asthma	NR
hsa-miR-376a-3p	miRNA	Homo sapiens	Asthma	NR
hsa-miR-423-3p	miRNA	Homo sapiens	Asthma	0.999999
hsa-miR-483-3p	miRNA	Homo sapiens	Asthma	0.998598
hsa-miR-485-3p	miRNA	Homo sapiens	Asthma	0.994838
hsa-miR-495-3p	miRNA	Homo sapiens	Asthma	NR
hsa-miR-508-3p	miRNA	Homo sapiens	Asthma	0.999141
hsa-miR-590-5p	miRNA	Homo sapiens	Asthma	1
hsa-miR-625-5p	miRNA	Homo sapiens	Asthma	0.973352
hsa-miR-629-3p	miRNA	Homo sapiens	Asthma	0.999141
hsa-miR-98-5p	miRNA	Homo sapiens	Asthma	0.999141

The table now contains one row for each of the 63 unique extracellular miRNAs listed in Table 1. Duplicate rows in the RNADisease strong-evidence batch export were collapsed by exact mature-miRNA name. NR, not returned by the strong-evidence batch filter at the time of extraction; NR does not imply absence of an asthma association in the broader RNADisease resource.

For analytic provenance, score-100 miRDB v6.0 targets were available for 34/63 candidates. Gene Ontology molecular-function analysis using the 2026 GO release identified zinc-ion binding (raw *p* = 4.07 × 10⁻^6^; FDR = 2.09 × 10⁻^2^). This legacy result is retained only for provenance because it is not bias-aware, was generated from a narrower score-100 target set, and is not biologically interpretable as evidence that zinc status, zinc binding, or zinc supplementation has a causal or therapeutic role in asthma.

The exploratory miRPathDB binary map was removed from the main manuscript, and the earlier score-100 network is retained only as provenance in the Supplementary Data workbook. Supplementary Figure S1 summarizes the corrected auditable workflow. These exploratory outputs are not used to estimate hub degree, select Reactome pathways, or determine statistical significance in the main analysis.

### 3.2 Literature-based concordance and screening audit

The retained auditable screening set comprised 23 records entering detailed review (7 repository records and 16 publication records). Three linked repository-publication pairs were consolidated, yielding 20 study-level units. Six units were excluded and 14 were retained in the evidence audit: one public-dataset published-result assessment, three formal literature-concordance studies, and ten contextual studies. GSE280322/PRJNA1177844 with its associated publication (11) was retained as the only high-throughput public dataset suitable for published-result concordance assessment. The dataset comprises 35 human plasma-derived EV ncRNA-sequencing samples across healthy controls, non-severe asthma, and severe asthma, corresponding to approximately 11–12 participants per group; the limited sample size may have reduced power, although differences in platform, contrasts, and cohort characteristics may also have contributed to the absence of exact overlap.

A *de novo* sample-level reanalysis of GSE280322 was feasible in principle but was not undertaken in the present study. Instead, the differentially abundant miRNAs reported for the comparison-specific contrasts in the associated publication were compared with the 63-miRNA panel using exact mature-arm matching. No exact match was observed. hsa-miR-133a-3p was not treated as equivalent to the panel's arm-unspecified legacy identifier hsa-miR-133b. This result is reported as published-result concordance and not as sample-level validation.

Three separate published human extracellular-miRNA studies met the formal exact mature-arm concordance rule (Table 3) (13–18). Vázquez-Mera et al. (14) supported hsa-miR-126-3p and hsa-miR-146a-5p; Vorobeva et al. (15) supported hsa-miR-223-3p, hsa-miR-191-5p, and hsa-miR-125b-5p; and Bahmer et al. (17) supported hsa-miR-122-5p. This yields six formally concordant candidates. Re-audit of the identifier nomenclature showed that Atashbasteh et al. (16) reported miR-124, miR-125b, miR-133b, and miR-130a without mature-arm resolution for the significant comparisons, while Rostamzadeh Khoie et al. (18) reported miR-155 and miR-221 without -3p/-5p resolution. These reports therefore provide only arm-unspecified contextual evidence and cannot be claimed as exact matches to hsa-miR-124-3p, hsa-miR-130a-3p, hsa-miR-155-3p, or hsa-miR-221-3p. Four additional panel candidates thus have contextual, but not exact-arm, support.

**Table 3:** Formal literature-based exact-arm concordance and arm-unspecified contextual evidence for the 63-miRNA candidate panel.

Study/dataset	Specimen and design	Literature-based result assessed	Exact overlap with current panel	Interpretation
Prasad et al. (11); GSE280322/PRJNA1177844; 35 samples	Plasma-derived EV ncRNA-seq; HC, non-severe asthma, severe asthma	Comparison-specific differentially abundant miRNAs displayed in the accession-associated publication	None by exact mature-arm matching	Potentially suitable public dataset; negative published-result exact-arm concordance. Raw-read reprocessing was feasible in principle but was not undertaken; not sample-level validation. The limited sample size (∼11–12/group) may have reduced power, although platform, contrast, and cohort differences may also have contributed to the absence of exact overlap.
Vázquez-Mera et al. (14); 30 healthy donors and 119 asthma patients	Serum exosomes; targeted RT-qPCR	Severity/phenotype-associated inflamma-miRNAs	hsa-miR-126-3p; hsa-miR-146a-5p	Formal exact mature-arm concordance in a separate published human extracellular-miRNA study.
Vorobeva et al. (15); 50 controls, 50 mild asthma, 50 severe asthma	Serum; targeted RT-qPCR panels	Diagnostic and severe-asthma-risk panels	hsa-miR-223-3p; hsa-miR-191-5p; hsa-miR-125b-5p	Formal exact mature-arm concordance in a separate published human extracellular-miRNA study.
Atashbasteh et al. (16); 30 severe asthma patients and 30 healthy controls	Plasma exosomes; targeted RT-qPCR	Significant group-level results were reported for miR-124, miR-125b, miR-133b, and miR-130a without mature-arm resolution; miR-125b-1-3p was not significantly different.	No exact mature-arm match assigned; arm-unspecified contextual correspondence to hsa-miR-124-3p, hsa-miR-125b-5p, hsa-miR-130a-3p, and legacy hsa-miR-133b.	Contextual only. Arm-unspecified names were not mapped to a specific -3p/-5p panel member and were not counted in the formal-concordance set.
Bahmer et al. (17); plasma EV RNA-seq asthma cohort	Plasma EVs; RNA-seq-based profiling	EV-miRNA signals reported in asthma, including miR-122-5p	hsa-miR-122-5p	Formal exact mature-arm concordance for hsa-miR-122-5p in a separate published plasma-EV asthma study; not sample-level validation of the complete panel.
Rostamzadeh Khoie et al. (18); 18 moderate-to-severe asthma patients and 18 healthy controls	Serum exosomes; targeted RT-qPCR	Significantly increased exosomal miR-155 and miR-221 were reported, but the publication used arm-unspecified identifiers.	No exact mature-arm match assigned; arm-unspecified contextual correspondence to hsa-miR-155-3p and hsa-miR-221-3p.	Contextual only. The arm-unspecified identifiers were not counted as exact mature-arm concordance.

### 3.3 Expanded predicted miRNA–target–pathway network

Of the 63 candidates, 59 mapped to 57 unique MIR:MIRDB target sets; hsa-miR-126-3p, hsa-miR-1307-3p, hsa-miR-187-3p, and hsa-miR-423-3p were not represented. Thus, the literature-concordance set and the network-analysis set were explicitly non-identical, and one formal concordance candidate (hsa-miR-126-3p) was included in literature-based concordance but absent from the MIR:MIRDB network. The mapped sets collectively contained 7,092 unique target genes. Counting each unique target set once, the primary analysis identified 108 hubs with degree ≥7. CLOCK and ZBTB20 had the highest degree (14 unique target sets each), followed by QKI (12), and BRWD3, GAB1, SESN3, SESTD1, and ZNF704 (11 each). Because hub status in predicted-target networks can be inflated by long, highly conserved 3′ UTRs and large predicted binding-site counts, these hubs are interpreted as network-prioritization outputs rather than asthma-central genes. The candidate-level sensitivity analysis, which counts jointly represented miRNAs separately, yielded 154 hubs with degree ≥7 and was not used as the primary estimate.

**Figure 1:**
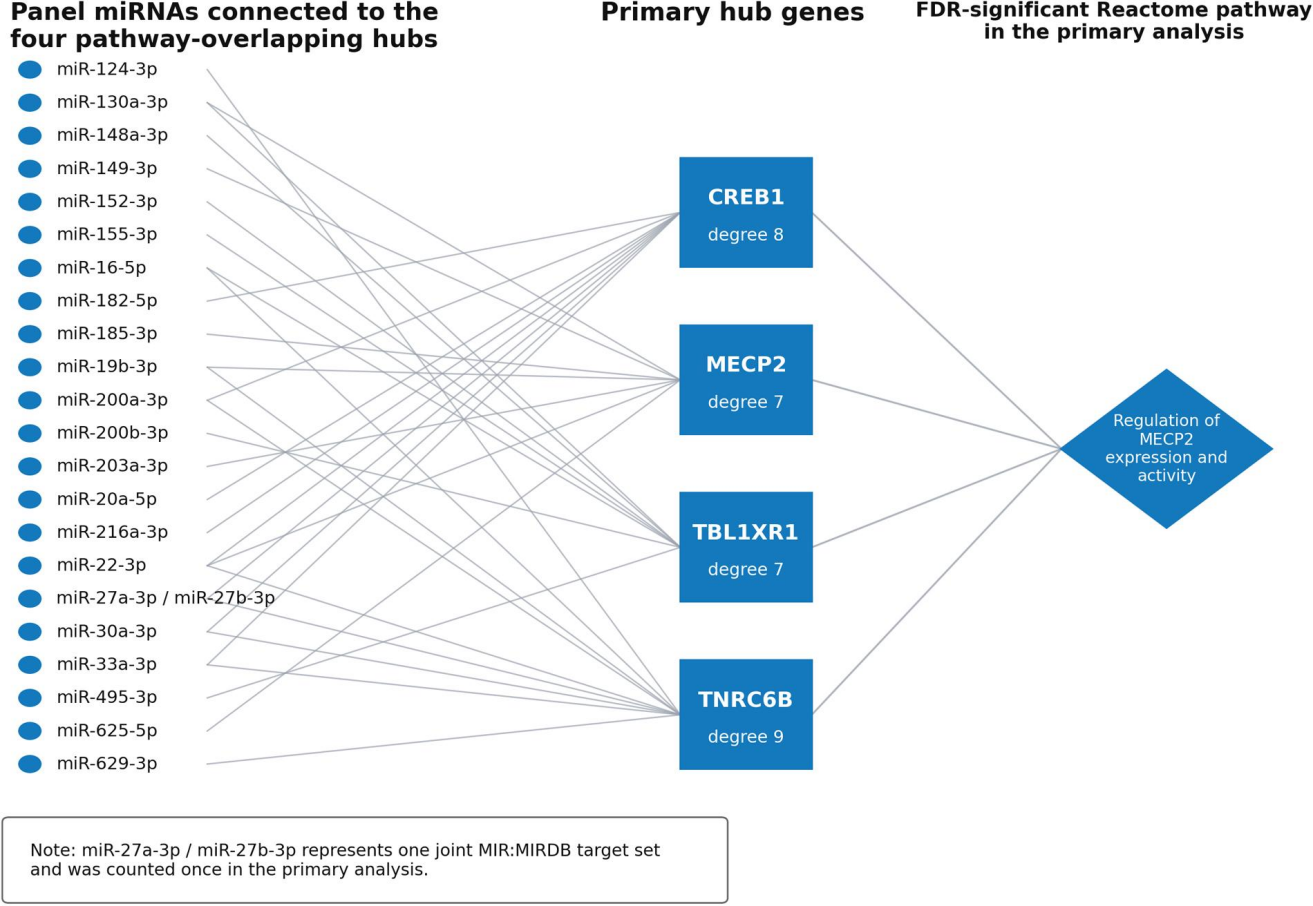
Duplicate-aware tripartite network for the primary analysis. Only panel miRNAs connected to at least one of the four pathway-overlapping primary hubs are displayed. Edges from miRNAs to CREB1, MECP2, TBL1XR1, and TNRC6B represent membership in unique MIR:MIRDB target sets; gene-to-pathway edges indicate membership in the Reactome term “Regulation of MECP2 Expression and Activity,” the only pathway significant at FDR < 0.05 in the primary unique-set analysis at degree ≥7. miR-27a-3p and miR-27b-3p are represented by one combined node because they correspond to a joint MIR:MIRDB target set and were counted once in the primary analysis. Hub labels report unique-target-set degrees (CREB1, 8; MECP2, 7; TBL1XR1, 7; TNRC6B, 9). The hsa- prefix is omitted from node labels for readability. The visualization is descriptive and does not establish binding, directionality, or activity in asthma-relevant cells.

Reactome analysis tested all 1,217 pathways passing the analysis-defined 10–500-gene size filter before multiplicity correction. In the primary unique-target-set analysis at degree ≥7, one pathway remained significant: regulation of MECP2 expression and activity (4/29 genes; CREB1, MECP2, TBL1XR1, and TNRC6B; raw *p* = 3.49 × 10⁻^5^; Benjamini–Hochberg FDR = 0.0425) (Table 4; Figure 1). Sensitivity analyses showed four significant pathways at degree ≥6 and none at degree ≥8. Candidate-level counting produced 22 significant pathways at degree ≥6 but none at degree ≥7 or ≥8, demonstrating that the MECP2 result is contingent on the analysis-defined hub threshold and counting unit. The inclusion of TNRC6B in the significant pathway, and the recurrence of AGO/TNRC6 genes in lower-threshold sensitivity outputs, indicates a partly self-referential contribution of the miRNA-silencing machinery that should be interpreted cautiously.

**Table 4:** Primary Reactome over-representation result after unique-target-set counting and Benjamini–Hochberg correction across all 1,217 eligible pathways.

Reactome pathway	Overlapping primary hubs	Overlap (hub/pathway)	Raw *p* value	Benjamini–Hochberg FDR
Regulation of MECP2 expression and activity	CREB1; MECP2; TBL1XR1; TNRC6B	4/29	3.49 × 10−5	0.0425

## 4 Discussion

Rather than simply aggregating database entries, the revised analysis separates candidate generation, formal exact mature-arm literature-based concordance, arm-unspecified contextual evidence, and mechanistic prioritization within a deliberately conservative framework. Three published human extracellular-miRNA studies provided formal exact mature-arm support for six candidates, whereas four additional candidates had only arm-unspecified contextual support. The published comparison-specific differential-miRNA results for GSE280322 showed no exact mature-arm overlap. This mixed pattern is biologically plausible given differences in biospecimen processing, EV isolation, untargeted vs. targeted platforms, phenotype definitions, statistical thresholds, and sample size, and it demonstrates why database-derived candidates should not be presented as a universally replicated or asthma-specific signature.

The absence of exact mature-arm overlap with the published GSE280322 differential-miRNA lists is informative but should not be over-interpreted. The dataset contains only 35 samples across three groups, corresponding to approximately 11–12 participants per group. Limited power may have contributed to the absence of overlap, although differences in platform, preprocessing, contrasts, statistical selection thresholds, and cohort characteristics may also have influenced the result. The strict matching rule prevents transferring evidence across opposite arms or related family members; for example, hsa-miR-133a-3p in GSE280322 does not validate hsa-miR-133b. The two arm-unspecified legacy identifiers were removed in sensitivity analysis, which preserved the single significant Reactome pathway but reduced the hub set at degree ≥7 from 108 to 96. Because RNADisease curates published literature, and because some literature-concordance studies may also contribute directly or indirectly to curated disease associations, literature concordance is best viewed as a prioritization signal rather than independent replication. This circularity risk is now explicitly acknowledged.

An important contribution of the present work is methodological rather than purely biological. Counting joint MIR:MIRDB target sets once reduced the degree ≥7 hub set from 154 under candidate-level counting to 108, and Benjamini–Hochberg correction across all 1,217 eligible pathways reduced broad pathway claims to one FDR-significant result: regulation of MECP2 expression and activity. This pathway connects CREB1, MECP2, TBL1XR1, and TNRC6B and provides a testable transcriptional-regulatory hypothesis; however, its significance was not preserved across all sensitivity settings. Hub genes such as CLOCK, ZBTB20, and QKI may be favored by long 3′ UTRs and high predicted binding-site counts, and the presence of TNRC6B, with AGO/TNRC6 recurrence in lower-threshold sensitivity analyses, indicates a partly self-referential enrichment of the miRNA-silencing machinery. Future work should therefore incorporate bias-aware checks, such as degree normalization for predicted-site burden or comparison with length-matched null models, before assigning disease centrality to these network outputs.

Several candidates have functional evidence consistent with broader inflammatory or remodeling modules, but this evidence does not confer asthma specificity. hsa-miR-20a-5p has been reported to attenuate allergic inflammation by targeting HDAC4 (19). hsa-miR-22-3p has been linked to HDAC6-dependent inflammatory signaling in macrophages (20) and to the NLRP3-caspase-1-IL-1β axis in a murine asthma model (21). hsa-miR-326 regulates profibrotic TGF-β-related genes in pulmonary fibrosis models (22). The six formally concordant candidates also require caution: hsa-miR-146a-5p, hsa-miR-223-3p, hsa-miR-126-3p, and hsa-miR-125b-5p are recurrently reported in inflammatory and vascular contexts; hsa-miR-122-5p is a liver-enriched circulating marker widely studied in hepatic injury (23); and hsa-miR-191-5p has been used as an endogenous serum/plasma qPCR reference because of its relative stability (24). Accordingly, recurrence across cohorts may reflect tissue injury, abundance, stability, assay popularity, or general inflammatory biology rather than asthma-specific pathogenicity.

From a clinical perspective, the present results support staged prioritization rather than immediate application. Before prospective evaluation as biomarkers, the candidates should undergo disease-specificity assessment against other inflammatory, allergic, infectious, and cardiometabolic conditions in which many of these miRNAs are also reported. A future validation study should use prospectively collected, well-phenotyped asthma cohorts; prespecified biospecimens; standardized EV isolation and quality-control procedures; hemolysis assessment; spike-in and endogenous normalization strategies; locked analytical thresholds; and an external multicenter validation cohort. Diagnostic, endotype, exacerbation, and treatment-response outcomes should be evaluated separately, with comparison against established clinical variables and assessment of incremental discrimination and calibration.

Rather than proposing a clinically validated miRNA signature, this study establishes a transparent and sensitivity-tested framework for distinguishing formally concordant candidates, arm-unspecified contextual signals, and parameter-contingent network hypotheses from findings that depend on database curation, target-set duplication, or analytic thresholds. The six formally concordant candidates, the four additional candidates with arm-unspecified contextual support, and the network-central miRNAs and genes therefore constitute a transparently classified set for disease-specificity testing and prospective experimental evaluation; none should yet be considered a diagnostic, prognostic, predictive, asthma-specific, or therapeutically actionable biomarker.

Major strengths of this study are the explicit separation of candidate generation, formal exact mature-arm literature-based concordance, arm-unspecified contextual evidence, and mechanistic prioritization; exact mature-arm matching that avoids inappropriate equivalence across opposite arms, arm-unspecified names, or related family members; harmonization to miRBase v22.1 nomenclature; unique-target-set counting that prevents duplicate inflation; Benjamini–Hochberg correction across all 1,217 eligible Reactome pathways; and sensitivity analyses across hub thresholds, counting units, and arm-annotation assumptions. The study also reports negative concordance and non-significant pathway results rather than selectively emphasizing supportive findings. The Supplementary Appendix and Supplementary Data workbook provide the search strategies, the retained-set screening flow, record-level screening and deduplication logs, identifier-resolution status, candidate and concordance tables, all tested pathways, and sensitivity outputs, thereby creating an auditable framework that can be extended in future prospective validation studies.

The study remains dependent on database-derived evidence and target prediction. RNADisease entries may be affected by curation, publication, language, and source-study duplication bias. A manual source-overlap audit was attempted for the six formally concordant candidates, but complete candidate-level RNADisease source-study provenance was not available in the accessible export; independence from the concordance studies therefore could not be established for any candidate. Association scores do not provide standardized effect sizes or expression direction. Extracellular-localization annotations are permissive, context dependent, and do not identify whether a miRNA is EV-associated, Ago2- or HDL-bound, passively released, or derived from a specific cell type. GSE280322 was identified as potentially suitable for sample-level assessment, and raw-read reprocessing through a standard small-RNA pipeline was feasible in principle; however, *de novo* sample-level differential-expression analysis was not undertaken in the present study. The reported GSE280322 assessment is limited to exact-arm concordance with published contrast-specific results and does not constitute sample-level validation. The formal concordance and contextual studies differ in specimen processing, EV definition, platform, candidate selection, age, asthma definition, and severity classification. The search audit reports the retained screening flow, but original raw interface hit totals and complete exports were not retained and are not retrospectively reconstructed. Target predictions depend on historical database releases retained to reproduce the completed network analysis. Hub thresholds were operational and analysis-defined during revision, and sensitivity analyses confirmed substantial variation in hub and pathway counts. hsa-miR-133b and hsa-miR-326 are legacy arm-unspecified identifiers. Finally, target prediction does not establish binding, directionality, cell specificity, or causal relevance, and RT-qPCR confirmation, rigorous EV characterization, longitudinal replication, bias-aware network analysis, and functional perturbation remain necessary.

## 5 Conclusion

A transparent multi-resource workflow identified 63 extracellular asthma-associated miRNA candidates and evaluated them using exact mature-arm concordance, duplicate-aware target-set counting, Benjamini–Hochberg correction across all 1,217 eligible pathways, and sensitivity analyses. Three published human extracellular-miRNA studies provided formal exact mature-arm concordance for six candidates, while four additional candidates had only arm-unspecified contextual support. Comparison with the published high-throughput results for GSE280322 showed no exact panel overlap and did not constitute sample-level validation. Duplicate-aware unique-target-set counting identified 108 hubs with degree ≥7 and one Reactome pathway after valid multiple-testing correction, but this pathway was parameter-contingent and partly influenced by predicted-target and miRNA-silencing machinery structure. The principal contribution is therefore a rigorous and auditable prioritization framework that identifies a transparently classified set for disease-specificity testing and prospective experimental evaluation, together with a parameter-dependent transcriptional-regulatory hypothesis requiring bias-aware and experimental confirmation.

## Data Availability

The raw data supporting the conclusions of this article will be made available by the authors, without undue reservation.
