# Supplementary material for: Prioritizing extracellular miRNA candidates in asthma: transparent in silico integration, literature-based concordance, and mechanistic network analysis
Source: Front Allergy. 2026 Aug 5;7:1856862. doi: 10.3389/falgy.2026.1856862 (PMC13485710; doi:10.3389/falgy.2026.1856862)
Supplement: Supplementary file 1 [file Datasheet1.zip › 1856862_Supplementary_Appendix_Final.docx]

Supplementary Appendix

Manuscript: “Prioritizing Extracellular miRNA Candidates in Asthma: Transparent In Silico Integration, Literature-Based Concordance, and Mechanistic Network Analysis”

Ourania S. Kotsiou; Irene Tsilioni; Nikolaos A. A. Balatsos; Konstantinos I. Gourgoulianis; Zoe Daniil; Erasmia Rouka

This concise appendix contains the methodological and analytical details needed to evaluate the revised manuscript: the auditable search strategy, eligibility and deduplication rules, the retained-set screening flow and identifier-resolution audit, the GSE280322 published-result assessment, the corrected MIR:MIRDB–Reactome workflow, and the primary sensitivity results. Complete record-level screening and deduplication logs, identifier-resolution classifications, candidate mappings, concordance results, and full pathway and sensitivity-analysis outputs are supplied separately in the Supplementary Data workbook.

S1. Search audit and counting convention

Searches were conducted on 12 June 2026. Raw live-interface hit totals and complete original exports were not retained and are not retrospectively reconstructed. Counts below therefore represent records entering the auditable detailed-screening or linkage-review set. The search is described as transparent and auditable rather than fully reproducible from inception. Linkage-only SRA/BioProject and mirror records used for accession verification are documented separately and are not counted as additional records in the 23-record detailed-review total.

Supplementary Table S1. Database-specific search strategies and auditable screening counts.

| **Database** | **Date** | **Exact search string** | **Auditable count** | **Counting note** |
| --- | --- | --- | --- | --- |
| NCBI GEO DataSets | 12 June 2026 | ((asthma OR "bronchial asthma") AND (miRNA OR microRNA) AND (serum OR plasma OR blood OR sputum OR saliva OR exosome OR "extracellular vesicle" OR "extracellular RNA")) | 7 GEO Series records entered detailed screening | Exact auditable screening count; not presented as the raw database total. |
| NCBI SRA / BioProject | 12 June 2026 | ((asthma OR "bronchial asthma") AND (miRNA OR microRNA) AND (serum OR plasma OR blood OR sputum OR saliva OR exosome OR "extracellular vesicle" OR "extracellular RNA")) | 3 linked SRA/BioProject records entered linkage review | Linked records were consolidated with the corresponding GEO Series and publications. |
| EMBL–EBI BioStudies / ArrayExpress | 12 June 2026 | asthma AND (miRNA OR microRNA) AND (serum OR plasma OR blood OR sputum OR saliva OR exosome OR "extracellular vesicle" OR "extracellular RNA") | 3 linked/mirrored records; 0 additional unique eligible datasets | Mirror and literature records were checked for accessions and downloadable matrices. |
| PubMed/MEDLINE | 12 June 2026 | ((asthma[Title/Abstract] OR "bronchial asthma"[Title/Abstract]) AND (miRNA[Title/Abstract] OR microRNA*[Title/Abstract]) AND (serum[Title/Abstract] OR plasma[Title/Abstract] OR blood[Title/Abstract] OR sputum[Title/Abstract] OR saliva[Title/Abstract] OR exosome*[Title/Abstract] OR "extracellular vesicle*"[Title/Abstract] OR "extracellular RNA"[Title/Abstract])) | 16 articles entered detailed screening | Complete bibliographic details of all records in the auditable set are supplied in Supplementary Data. |
| Europe PMC | 12 June 2026 | ((TITLE:asthma OR ABSTRACT:asthma OR TITLE:"bronchial asthma" OR ABSTRACT:"bronchial asthma") AND (TITLE:miRNA OR ABSTRACT:miRNA OR TITLE:microRNA OR ABSTRACT:microRNA) AND (serum OR plasma OR blood OR sputum OR saliva OR exosome OR "extracellular vesicle" OR "extracellular RNA")) | 16 overlapping articles entered cross-checking | Used to verify full text, accessions, and supplementary-data availability; deduplicated against PubMed. |

S2. Eligibility, screening, and deduplication

- Population: human participants with asthma or a clearly defined asthma phenotype/severity group and an appropriate comparator.
- Specimen: serum, plasma, cell-free blood fraction, saliva, sputum supernatant, purified exosomes/extracellular vesicles, or another explicitly extracellular RNA fraction.
- Assay: mature-miRNA profiling with arm-resolved identifiers where applicable.
- Public-dataset assessment eligibility: public sample-level expression data or raw reads plus group/phenotype annotations.
- Formal literature-concordance eligibility: a separate published human extracellular or cell-free miRNA study reporting an exact mature-arm-resolved -3p/-5p identifier, a clearly defined asthma comparator/phenotype/severity contrast, and a direction-specific or significance-specific group-level result. A reusable sample-level matrix was not required for this category.
- Contextual evidence: studies using arm-unspecified miRNA names, reporting family-level identifiers, or lacking sufficient candidate-level information for exact mature-arm matching. Arm-unspecified names were not mapped to a specific -3p/-5p panel member.

Deduplication was performed by GEO/SRA/BioProject accession, PMID, DOI, and normalized title. Linked GEO–SRA/BioProject–publication records were treated as one study-level unit. The retained audit comprised 23 records entering detailed review (7 repository records and 16 publication records). Consolidation of three linked repository–publication pairs yielded 20 study-level units; 6 were excluded and 14 were retained in the evidence audit (1 public-dataset published-result assessment, 3 formal literature-concordance studies, and 10 contextual studies). Complete record-level dataset, article, linkage, exclusion, identifier-resolution, and final-classification logs are provided in the Supplementary Data workbook.

S3. GSE280322 published-result assessment

GSE280322/PRJNA1177844 and its associated publication report plasma-derived extracellular-vesicle ncRNA sequencing in 35 samples spanning healthy controls, non-severe asthma, and severe asthma. GEO provides a normalized-count matrix and links to raw SRA reads. A de novo sample-level differential-expression reanalysis was feasible in principle but was not undertaken. Valid raw-read reprocessing would require a newly specified small-RNA pipeline, including adapter trimming, reference-miRNA release selection, alignment, feature quantification, quality-control thresholds, and normalization decisions. Normalized values were therefore not rounded, converted into pseudo-counts, or analyzed as raw counts.

Exact mature-miRNA identifiers reported as differentially abundant for the comparison-specific contrasts were compared with the 63-miRNA panel. No exact match was observed. hsa-miR-133a-3p was not considered equivalent to the panel’s legacy arm-unspecified hsa-miR-133b. This assessment is reported as published-result concordance and not as sample-level validation or independent replication.

S4. Corrected MIR:MIRDB–Reactome workflow

1. Use the 63 unique candidates and preserve mature-arm identifiers.
2. Map candidates to MSigDB v7.4 C3 MIR:MIRDB target sets; flag hsa-miR-133b and hsa-miR-326 as legacy arm-unspecified identifiers.
3. Count the joint MIR27A_3P_MIR27B_3P and MIR29B_3P_MIR29C_3P target sets once in the primary analysis.
4. Map 59 candidates to 57 unique target sets and form the union of 7,092 target genes.
5. Define primary hubs operationally as genes targeted by at least seven unique target sets; examine thresholds ≥6 and ≥8 in sensitivity analyses.
6. Use the 16,668 MIR:MIRDB genes as background and apply the analysis-defined Reactome pathway-size filter of 10–500 background genes.
7. Calculate one-sided hypergeometric p values for all 1,217 eligible pathways and apply Benjamini–Hochberg correction across the full family.
8. Repeat candidate-level counting and repeat the primary analysis after excluding hsa-miR-133b and hsa-miR-326.

S5. Boundary of exploratory analyses

The exploratory binary miRPathDB map was removed from the main manuscript because it was non-inferential and its exact versioned export was not preserved sufficiently for reliable reproduction. Its provenance and interpretive boundary, together with the earlier score-100 miRDB/Gene Ontology and multiple-query network outputs, are retained in the Supplementary Data workbook only. They were not used for primary hub definition, Reactome testing, or FDR-controlled conclusions. The corrected unique-target-set MIR:MIRDB–Reactome workflow is the sole primary inferential network analysis. Supplementary Figure S1 summarizes the corrected auditable search, linkage, eligibility, evidence-classification, published-result, and network-analysis workflow.

S6. Primary and sensitivity results

Supplementary Table S2. Corrected primary and sensitivity-analysis results.

| **Analysis** | **Counting unit** | **Hub threshold** | **Targets** | **Hubs** | **Eligible pathways** | **FDR-significant pathways** |
| --- | --- | --- | --- | --- | --- | --- |
| Primary | Unique MIR:MIRDB target set | ≥7 | 7,092 | 108 | 1,217 | 1 |
| Sensitivity | Unique target set | ≥6 | 7,092 | 221 | 1,217 | 4 |
| Sensitivity | Unique target set | ≥8 | 7,092 | 48 | 1,217 | 0 |
| Sensitivity | Candidate-level | ≥6 | 7,092 | 312 | 1,217 | 22 |
| Sensitivity | Candidate-level | ≥7 | 7,092 | 154 | 1,217 | 0 |
| Sensitivity | Candidate-level | ≥8 | 7,092 | 78 | 1,217 | 0 |
| Sensitivity excluding hsa-miR-133b and hsa-miR-326 | Unique target set | ≥7 | 6,978 | 96 | 1,217 | 1 |

The only FDR-significant pathway in the primary analysis was Regulation of MECP2 Expression and Activity (4/29 genes: CREB1, MECP2, TBL1XR1, and TNRC6B; raw p=3.49×10−5; Benjamini–Hochberg FDR=0.0425). This result was parameter-contingent: it was not significant at the ≥8 hub threshold or under candidate-level counting at ≥7. Broader pathway findings under alternative thresholds are reported as sensitivity results and are not treated as the primary biological conclusion.

Supplementary Figure S1

Corrected auditable search, study linkage, eligibility, identifier-resolution classification, literature-based assessment, and primary network-analysis workflow. Counts begin with records entering the retained detailed-review set and do not represent retrospectively reconstructed live-interface totals.

Supplementary Data workbook

The separate Supplementary Data workbook contains the complete screening and deduplication logs, the retained-set flow, identifier-resolution and final evidence classification for every screened study, the GSE280322 concordance table, candidate mappings, all 7,092 targets, all 108 primary hubs, all 1,217 tested pathways, and all sensitivity outputs. These records support audit of the analyses without expanding the inferential scope defined in this appendix.
